# Nebulized hypertonic saline triggers nervous system-mediated active liquid secretion in cystic fibrosis swine trachea

**DOI:** 10.1038/s41598-018-36695-4

**Published:** 2019-01-24

**Authors:** Xiaojie Luan, Julian S. Tam, George Belev, Santosh Jagadeeshan, Brendan Murray, Noman Hassan, Terry E. Machen, L. Dean Chapman, Juan P. Ianowski

**Affiliations:** 1 University of Saskatchewan, Department of Physiology, Health Science Building, Room 2D01, 107 Wiggins Rd., Saskatoon, Saskatchewan S7N 5E5 Canada; 2 University of Saskatchewan, Department of Medicine, Division of Respirology, Critical Care, and Sleep Medicine, Royal University Hospital, 103 Hospital Drive, Saskatoon, Saskatchewan S7N 0W8 Canada; 3 Canadian Light Source Inc., 44 Innovation Boulevard, Saskatoon, Saskatchewan S7N 2V3 Canada; 4 University of California, Department of Molecular and Cell Biology, 231 LSA, Berkeley, CA 94720-3200 USA; 5 University of Saskatchewan, Department of Anatomy and Cell Biology, Health Science Building, Room 2D01, 107 Wiggins Rd., Saskatoon, Saskatchewan S7N 5E5 Canada

## Abstract

Inhaled hypertonic saline (HTS) treatment is used to improve lung health in patients with cystic fibrosis (CF). The current consensus is that the treatment generates an osmotic gradient that draws water into the airways and increases airway surface liquid (ASL) volume. However, there is evidence that HTS may also stimulate active secretion of ASL by airway epithelia through the activation of sensory neurons. We tested the contribution of the nervous system and airway epithelia on HTS-stimulated ASL height increase in CF and wild-type swine airway. We used synchrotron-based imaging to investigate whether airway neurons and epithelia are involved in HTS treatment-triggered ASL secretion in CFTR^−/−^ and wild-type swine. We showed that blocking parasympathetic and sensory neurons in airway resulted in ~50% reduction of the effect of HTS treatment on ASL volume *in vivo*. Incubating tracheal preparations with inhibitors of epithelial ion transport across airway decreased secretory responses to HTS treatment. CFTR^−/−^ swine *ex-vivo* tracheal preparations showed substantially decreased secretory response to HTS treatment after blockage of neuronal activity. Our results indicated that HTS-triggered ASL secretion is partially mediated by the stimulation of airway neurons and the subsequent activation of active epithelia secretion; osmosis accounts for only ~50% of the effect.

## Introduction

Inhaled hypertonic saline (HTS) is a well-established treatment for patients with cystic fibrosis (CF) and patients with non-CF bronchiectasis^1,2^. HTS treatment has been shown to improve mucociliary clearance, forced expiratory volume in 1 s, frequency of exacerbations, days on antibiotics, and well-being^1–4^. Recent analyses of lung clearance index and spirometry data suggest that HTS treatment may be able to halt the progression of mild CF lung disease^5^. Though CFTR modulators have been shown to improve outcomes in individuals with certain CFTR gene mutations^6–9^, HTS is a mutation-agnostic treatment that benefits patients with CF regardless of genotype.

The exact mechanism of action of HTS is not understood^3^, which makes it difficult to develop procedures to improve treatment outcomes such as through modulating the duration and intensity of the treatment effect^10^. The current consensus understanding of the mechanism of action of HTS inhalation is that the treatment generates an osmotic gradient that draws water into the airways^11,12^. This increases the volume of airway surface liquid (ASL), which improves mucus rheological properties and accelerates mucus transport rates^3,4^. The intensity of treatment has been proposed to depend on the aquaporin-mediated water permeability of the airway epithelia cells^11,12^.

However, there is evidence that HTS may also stimulate sensory nerves in the airways, triggering ASL secretion by airway epithelia. In rat airways, treatment with HTS stimulates neurogenic inflammation, specifically through the local release of inflammatory mediators by sensory-efferent pathways^13–15^. In guinea pig airways, HTS treatment activates airway afferent nerves including Aδ-and C-fibers both *in vitro* and *in vivo*^16,17^. Stimulation of C- and Aδ-fibres causes local anterograde release (i.e. axon reflex), in addition to reflex mediated by the central nervous system, of neurotransmitters which trigger ASL secretion^18,19^. Treatment of the nasal cavity with HTS in healthy volunteers stimulates nociceptive nerves and glandular mucus exocytosis^20^. In addition, HTS induces robust reflex responses in the nose and larynx of guinea pigs, similar to those evoked by capsaicin, and evokes coughing when applied topically to the tracheal or laryngeal mucosa^21^. In ferret trachea, HTS stimulates the production of two markers of gland secretion, mucins and lysozyme^22^.

The goal of the present study was to test the role of airway neurons and epithelia in HTS-stimulated ASL production in the tracheas of wild-type and CFTR^−/−^ swine using a novel synchrotron-based imaging method, which we developed to measure and quantify ASL secretion in live animals and *ex vivo* trachea preparations^23–25^. Our results show that HTS-stimulated ASL height increase in both wild-type and CFTR^−/−^ swine is reduced by inhibiting either neuronal function or epithelial ion secretion into the ASL. These results suggest that approximately 50% of the ASL produced by HTS treatment in wild-type and CF airway is mediated by the activation of the nervous system and stimulation of active epithelial ASL secretion.

## Results

**Figure 1:**
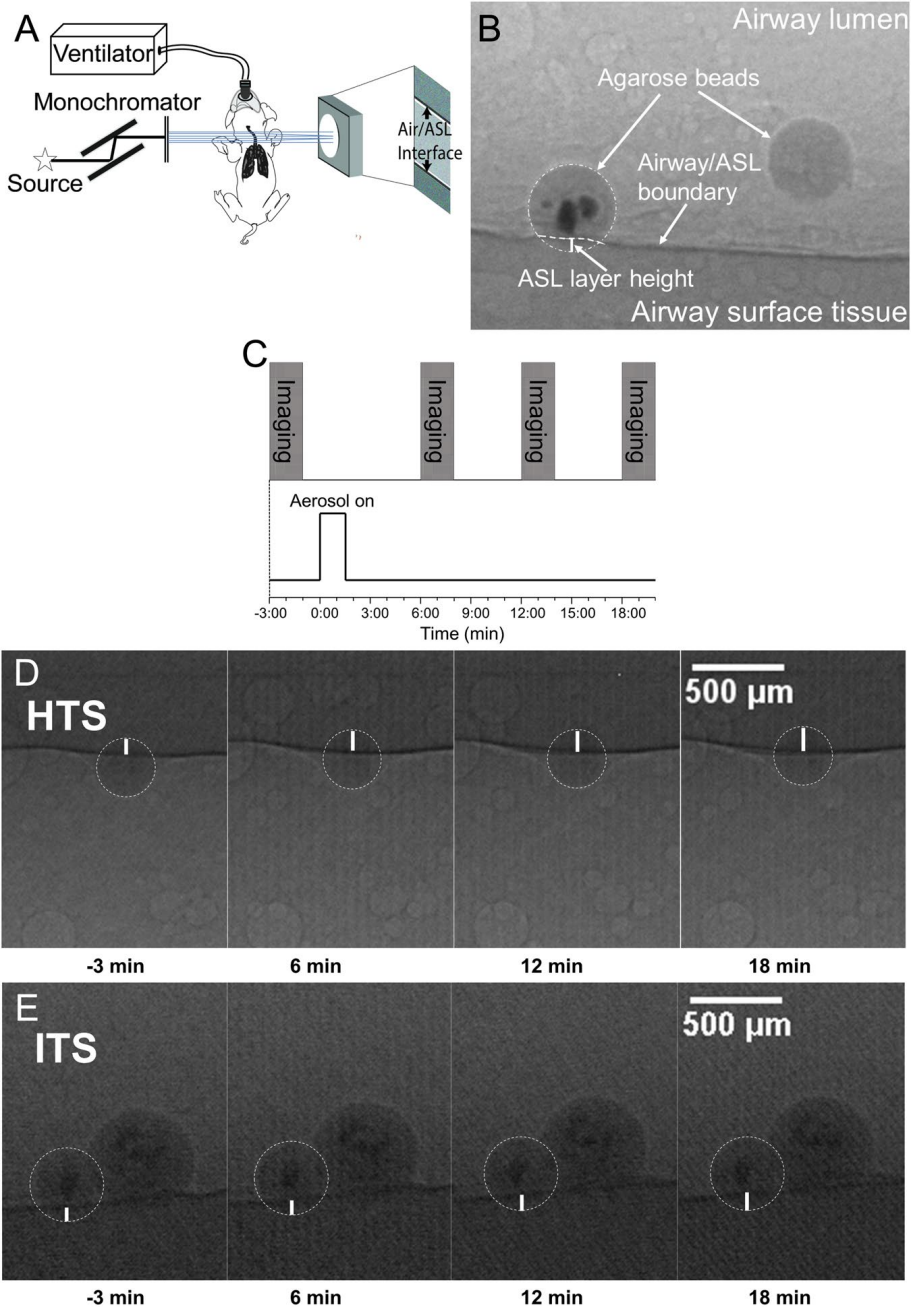
Experimental design and phase contrast imaging using synchrotron x-rays. (**A**) Schematic showing the set-up for ASL height measurement in the lumen of the trachea using phase contrast imaging. When x-rays pass through the preparation, the difference in refractive index between the ASL and the air results in a phase shift of x-rays that causes a distinct interference pattern detected as variations in x-ray intensities on the CCD (see^23^ for *ex vivo* diagram). (**B**) Synchrotron-based phase contrast imaging measurement of ASL height in an isolated swine trachea. (**C**) HTS or ITS aerosol were delivered at time 0 for 90 seconds, and images were acquired at time −3, 6, 12, and 18 minutes. Representative sample of the images acquired from an *ex vivo* preparation treated with (**D**) HTS and (**E**) ITS nebulization at −3, 6, 12, and 18 min.

**Figure 2:**
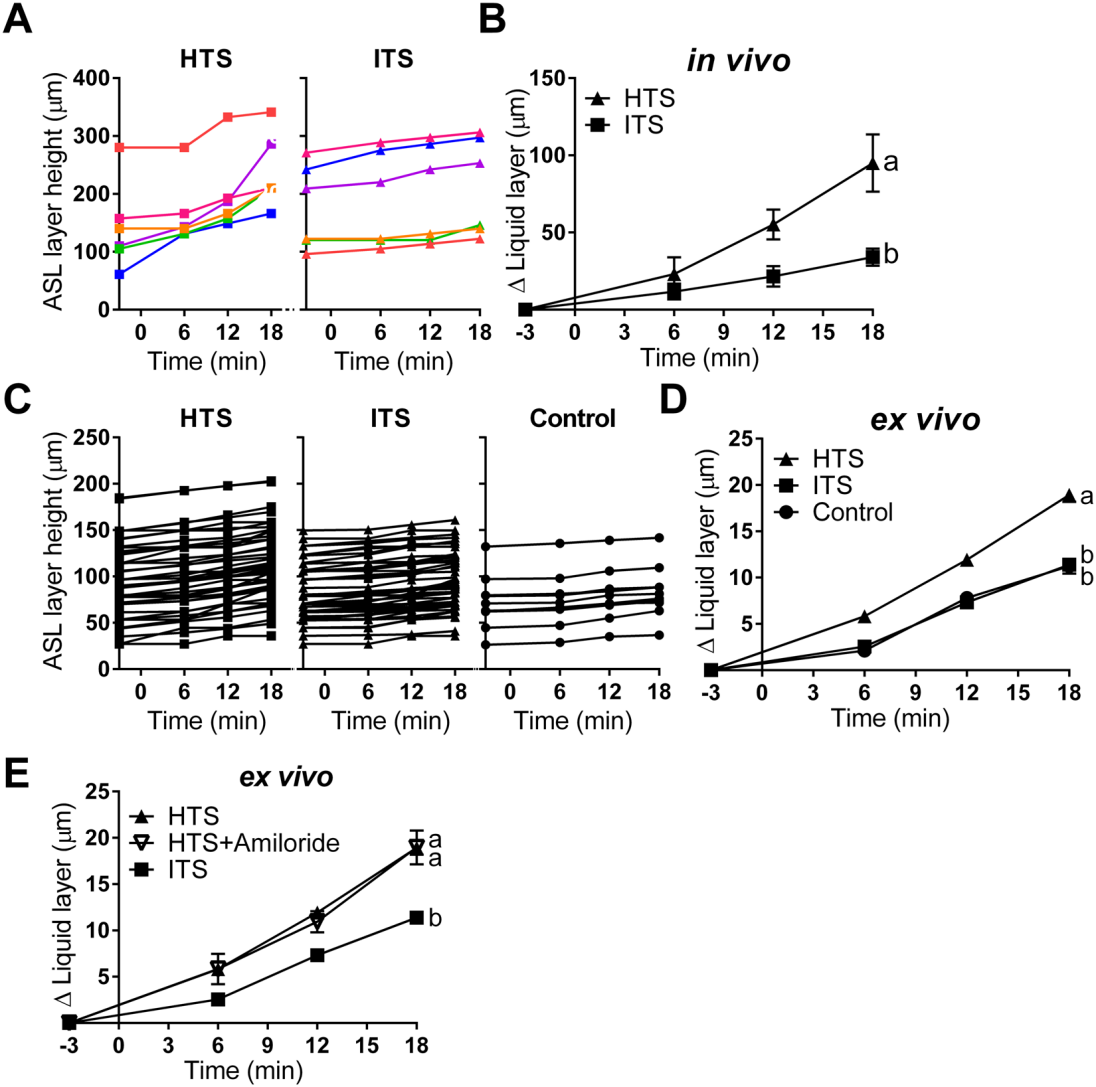
HTS triggers ASL secretion *in vivo* and *ex vivo* preparations. (**A**) scatter plot of HTS and ITS treatment on ASL height in live swine and (**B**) change in ASL height (HTS, n = 6 beads from 4 swine; ITS, n = 6 beads from 5 swine). (**C**) scatter plot of HTS and ITS treatment on ASL volume in *ex vivo* trachea preparation and (**D**) change in ASL height (HTS, n = 45 beads from 15 tracheas; ITS, n = 49 beads from 14 tracheas; control, n = 12 beads from 5 tracheas). (**E**) Amiloride did not affect the HTS treatment result (HTS, n = 45 beads from 15 tracheas; ITS, n = 49 beads from 14 tracheas; HTS + Amil, n = 12 beads from 5 tracheas). Data are presented as mean ± SEM and values at 18 min were analyzed with ANOVA and Tukey’s multiple comparison test. Data sets labeled with different letters differ significantly, p < 0.05.

We used a novel synchrotron-based imaging method to quantify ASL secretion and determine the height of the ASL layer, as described elsewhere^23,24^ (Fig. 1, see methods). Nebulized hypertonic (7% NaCl solution w/v) or isotonic (0.9% NaCl solution w/v) saline was administered to live wild-type swine (Fig. 1A)^2^. As expected, treating pigs (*in vivo*) with HTS significantly increased ASL height compared to preparations nebulized with isotonic saline (ITS) (Fig. 2A,B, Supplementary Table 1). A similar result was obtained from isolated tracheas (*ex vivo*), where HTS-treated preparations triggered greater ASL secretion than ITS-treated or untreated (control) ones (Fig. 2C,D). This suggests that HTS treatment does indeed increase ASL production in our preparations. To determine whether the ASL volume increase could be explained by the inhibition of ASL reabsorption by epithelial sodium channel (ENaC)-mediated pathway after HTS treatment^26^, we added the ENaC inhibitor, amiloride, to the HTS treatment. Our results showed that amiloride did not affect HTS treatment in the pig airway (Fig. 2E), which indicates that HTS-triggered increase in ASL layer height results from the production of liquid into the airway lumen, and not by blocking liquid absorption.

### HTS-triggered ASL height increase is partially mediated by the nervous system

Since previous reports indicated that HTS may stimulate sensory neurons and the autonomic nervous system^13,20^, we tested the effect of blocking the nervous system on HTS-triggered ASL height increase *in vivo*. Agarose beads were used both as “measuring rods” (see methods section) and as vehicles to deliver lidocaine into the trachea. Agarose beads have been used as vehicles for drug delivery^23,24^ in previous studies; Luan *et al*. showed that beads can be loaded with bacteria, LPS and flagellin and that these compounds leach out of the beads and stimulate submucosal gland ASL production in swine trachea^23,24^.

**Figure 3:**
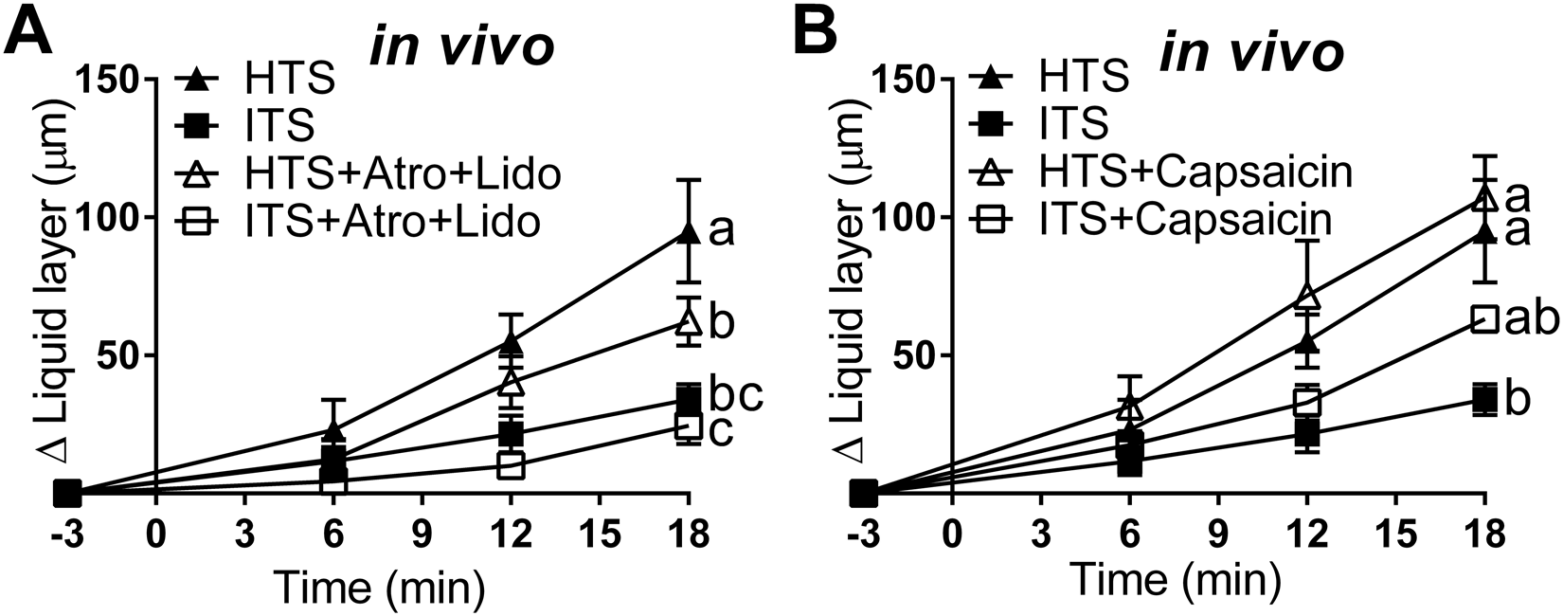
The nervous system contributes to HTS-triggered ASL secretion. (**A**) Atropine combined with lidocaine reduced HTS-triggered ASL secretion but had no effect in ITS-treated swine *in vivo* (HTS, n = 6 beads from 4 animals; ITS, n = 6 beads from 5 animals; HTS + Atro + Lido, n = 6 beads from 5 animals; ITS + Atro + Lido, n = 5 beads from 4 animals). (**B**) Stimulating C-fibers with capsaicin increased the secretion during ITS but not HTS treatment in live swine (HTS, n = 6 beads from 4 animals; ITS, n = 6 beads from 5 animals; HTS + Capsaicin, n = 6 beads from 4 animals; ITS + Capsaicin, n = 5 beads from 4 animals). Data are presented as mean ± SEM and values at 18 min were analyzed with ANOVA and Tukey’s multiple test. Data sets labeled with different letters differ significantly, p < 0.05.

Swine were treated with atropine (0.04 mg/kg, intramuscular)^27^ to block the autonomic nervous system, and the agarose beads were loaded with lidocaine (80 mg/ml)^28^ to inhibit sensory neurons in the airway. This treatment (HTS + Atro + Lido) reduced HTS-triggered (HTS) ASL height increase *in vivo* by ~50%. However, the atropine-plus-lidocaine treatment had no effect on ITS-treated swine (Fig. 3A, Supplementary Table 1). These results suggest that HTS treatment, but not ITS, recruits the nervous system. Approximately 50% of the HTS-triggered ASL production is mediated by stimulation of sensory neurons and autonomic nervous system.

Since HTS has been proposed to stimulate C-fibers^29^, which also respond to capsaicin, we tested whether capsaicin (10 µM) affected HTS treatment^30^. Agarose beads loaded with capsaicin (ITS + Cap) resulted in an increase in the ASL layer height greater than in swine treated with ITS (ITS) nebulization alone (Fig. 3B) indicating that C-fiber stimulation triggers ASL production. In contrast, combining capsaicin with HTS (HTS + Cap) treatment did not further increase HTS-triggered (HTS) ASL height (Fig. 3B), suggesting that HTS and capsaicin may act on the same target (i.e. C-fibers). Suggesting that activation of airway neurons contributes to fluid secreted into the airway during HTS nebulization.

**Figure 4:**
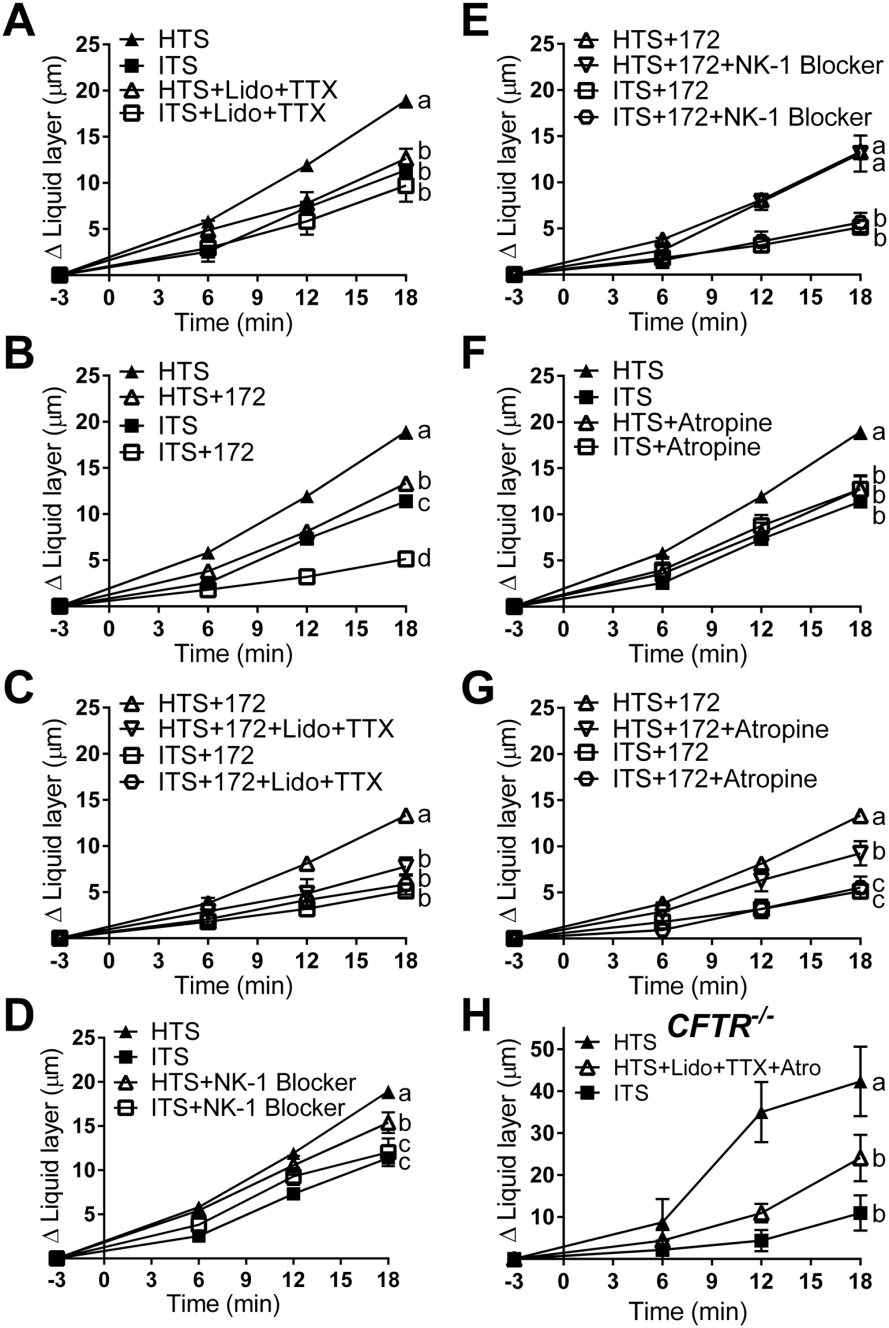
HTS stimulates *ex vivo* ASL secretion via activation of sensory neurons and release of acetylcholine in wild-type and CF airways. (**A**) Treatment with lidocaine (Lido, 80 mg/ml aerosol) plus tetrodotoxin (TTX, 1 µM) (HTS, n = 45 beads from 15 tracheas; ITS, n = 49 beads from 14 tracheas; HTS + Lido + TTX, n = 18 beads from 4 tracheas; ITS + Lido + TTX, n = 9 beads from 4 tracheas). (**B**) Incubation with CFTRinh172 (172, 100 µM) (HTS, n = 45 beads from 15 tracheas; ITS, n = 49 beads from 14 tracheas; HTS + 172, n = 59 beads from 12 tracheas; ITS + 172, n = 29 beads from 8 tracheas). (**C**) Incubation with lidocaine plus TTX on CFTRinh172-treated airway (HTS + 172, n = 59 beads from 12 tracheas; HTS + 172 + Lido + TTX, n = 9 beads from 4 tracheas; ITS + 172, n = 29 beads from 8 tracheas; ITS + 172 + Lido + TTX, n = 21 beads from 6 tracheas). (**D**) Effect of L-703606 (NK-1 blocker, 1 µM) (HTS, n = 45 beads from 15 tracheas; ITS, n = 49 beads from 14 tracheas; HTS + NK-1 blocker, n = 36 beads from 12 tracheas; ITS + NK-1 blocker, n = 16 beads from 5 tracheas). (**E**) Effect of L-703606 on CFTRinh172-treated preparations (HTS + 172, n = 59 beads from 12 tracheas; HTS + 172 + NK-1 blocker, n = 10 beads from 4 tracheas; ITS + 172, n = 29 beads from 8 tracheas; ITS + 172 + NK-1 blocker, n = 17 beads from 6 tracheas). (**F**) Effect of atropine (1 µM) (HTS, n = 45 beads from 15 tracheas; ITS, n = 49 beads from 14 tracheas; HTS + Atropine, n = 22 beads from 5 tracheas; ITS + Atropine, n = 11 beads from 4 tracheas). (**G**) Effect of atropine (1 µM) on CFTRinh172-treated preparations (HTS + 172, n = 59 beads from 12 tracheas; HTS + 172 + Atropine, n = 18 beads from 5 tracheas; ITS + 172, n = 29 beads from 8 tracheas; ITS + 172 + Atropine, n = 19 beads from 5 tracheas). (H) Effect of lidocaine, TTX, and atropine on CFTR^−/−^ swine trachea (HTS, n = 6 beads from 2 tracheas; HTS + Lido + TTX + Atro, n = 4 beads from 2 tracheas; ITS, n = 4 beads from 2 tracheas). Data are presented as mean ± SEM and values at 18 min were analyzed with ANOVA and Tukey’s multiple test. Data sets labeled with different letters differ significantly, p < 0.05.

To further test this idea, we used isolated pig trachea preparations for more invasive experiments. It has been established that incubating trachea preparations in tetrodotoxin (TTX, 1 µM) and lidocaine (10 mg) (single luminal application of 80 mg/ml spray)^28^ blocks both the voltage-dependent sodium channels expressed by neurons intrinsic to the airways^31,32^ and the TTX-independent sodium channels expressed by sensory neurons of the airways^18,33^ without directly altering epithelial ion secretion^24^. We found that blocking neuronal function with lidocaine + TTX reduced the effect of HTS treatment (Fig. 4A). In contrast, lidocaine + TTX had no effect on ITS-treated preparations, suggesting that the effect of lidocaine + TTX is specific to HTS treatment (Fig. 4A).

To evaluate the effect of HTS treatment in CF airways, we used the CFTR blocker CFTRinh172 (100 µM) in isolated wild-type trachea preparations to model airways without CFTR function^34^. HTS treatment in preparations incubated with CFTRinh172 (HTS + 172) was less effective in stimulating ASL height increase than preparations not incubated in CFTR blocker (HTS). However, preparations incubated with CFTRinh172 still produced greater ASL height increase when treated with HTS than ITS (Fig. 4B). This finding is consistent with previous experiments showing that HTS treatment improves ASL hydration in CF patients^35,36^. Interestingly, CFTRinh172 treatment reduced ASL secretion in ITS-treated samples (ITS + 172) below that of ITS treatment in control preparations (ITS, Fig. 4B), which may be explained by the blockage of CFTR-dependent basal ASL secretion in swine trachea as described in another study^24^.

Preparations incubated with CFTRinh172 suffered a reduction in the response to HTS treatment when neuronal function was blocked with lidocaine + TTX (Fig. 4C). In contrast, ITS-treated preparations were not affected by lidocaine + TTX (Fig. 4C). These results suggest that HTS treatment, but not ITS, increases ASL height through stimulation of sensory and/or airway intrinsic neurons in CF airways.

Since there is evidence that HTS treatment triggers substance P (SP) release^20^ and that SP can stimulate ASL secretion by airway epithelia in the trachea of several species^19,37,38^, we tested the effect of a SP receptor (neurokinin 1 receptor, NK-1) blocker, L-703606 (1 µM) on HTS-stimulated ASL height increase. Treatment with L-703606 (HTS + NK-1 Blocker) significantly reduced HTS-triggered ASL height increase, but had no effect on the ITS treatment group (ITS + NK-1 Blocker, Fig. 4D). However, HTS treatment of preparations incubated with CFTRinh172 was unaffected by the NK-1 blocker (Fig. 4E), indicating that the substance P-mediated HTS-triggered effect is CFTR-dependent and may be absent in CF airways.

We then tested the possible role of muscarinic stimulation on HTS-triggered ASL production. Blocking muscarinic receptors with atropine (1 µM)^38^ reduced HTS-triggered ASL secretion but had no effect on ITS treated samples (Fig. 4F). Atropine also reduced the response to HTS treatment in preparations incubated with the CFTR blocker CFTRinh172, but had no effect on ITS-treated preparations, suggesting a role for cholinergic signaling in HTS-triggered ASL secretion in both CF and non-CF airways.

To further test the contribution of the nervous system to HTS-triggered ASL secretion in CF, we studied the effect of HTS on CFTR^−/−^ swine trachea preparations (Fig. 4H). Treatment of CFTR^−/−^ tracheas with HTS significantly increased ASL height compared to ITS-treated preparations. The HTS effect was significantly reduced by treatment with blockers of the nervous system, lidocaine + TTX + atropine (Fig. 4H). These results further corroborate that HTS treatment stimulates airway neurons which release acetylcholine on airway epithelia and trigger active ASL secretion in CF airways.

### HTS treatment triggers active secretion of ASL by airway epithelia

To investigate the possible role of active epithelial ASL secretion in response to HTS treatment, we tested pharmacological agents that block epithelial ASL production in isolated trachea preparations. If HTS triggers neuron-stimulated epithelial ASL secretion, one would expect to block the effect of HTS by blocking ion transport by epithelial cells. However, if the effect of HTS is entirely mediated through osmosis, then an ion transport blocker treatment should not affect it. As most of the ASL in the upper airways (i.e. down to about the 10^th^ bronchial generation, where airway lumen is ~1–2 mm in diameter) is produced by submucosal glands^39^, we tested blockers known to abrogate gland secretions on HTS-triggered ASL production: the CFTR blocker CFTRinh172, the Na^+^:K^+^:2Cl^−^ cotransporter blocker bumetanide (100 µM)^39^, and a Ca^2+^-activated Cl^−^ channel blocker niflumic acid (100 µM)^36^ in HCO_3_^−^- free saline solution^40^.

**Figure 5:**
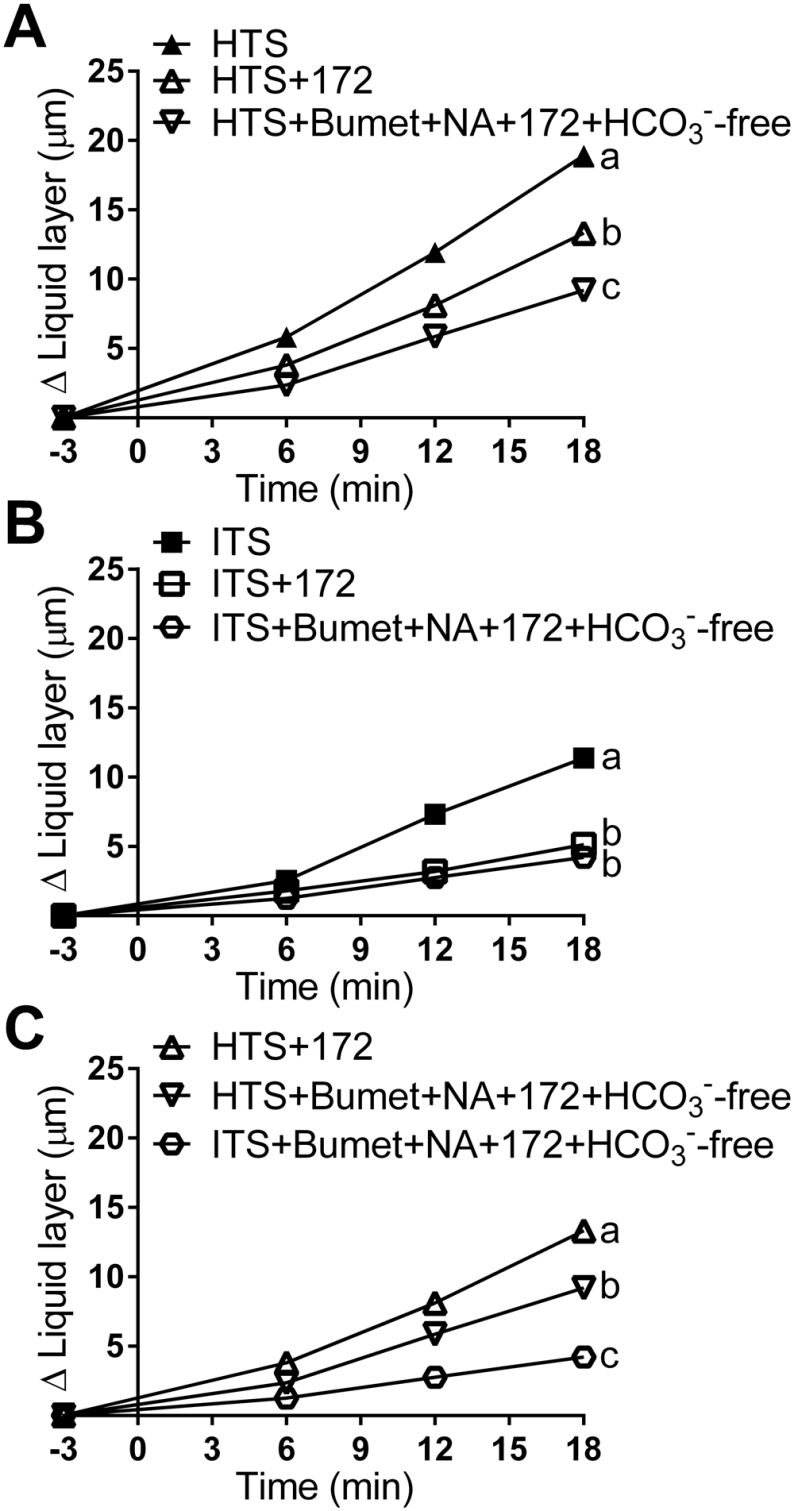
HTS treatments stimulate active ASL production by airway epithelia. (**A**) Treatment with the CFTR blocker CFTRinh172 (100 µM) reduced HTS-triggered ASL height increase, and treatment with CFTRinh172, bumetanide (100 µM), and niflumic acid (100 µM) in HCO_3_^−^-free saline solution bath reduced HTS-triggered ASL height increase even further (HTS, n = 45 beads from 15 tracheas; HTS + 172, n = 59 beads from 12 tracheas; HTS + Bumet + NA + 172 + HCO_3_^−^-free, n = 24 beads from 7 tracheas). (**B**) In ITS-treated preparations incubation with the ion transport blocker cocktail (CFTRinh172, bumetanide, and niflumic acid in HCO_3_^−^-free bath) had a similar effect as CFTRinh172 alone (ITS, n = 49 beads from 14 tracheas; ITS + 172, n = 29 beads from 8 tracheas; ITS + Bumet + NA + 172 + HCO_3_^−^-free, n = 18 beads from 7 tracheas). (**C**) Approximately 50% of the ASL produced by HTS in airways without CFTR function is the result of the osmotic effect. After blocking all ion transport with CFTRinh172, bumetanide, and niflumic acid in HCO_3_^−^-free saline, HTS produced ~50% less ASL secretion than that produced by preparations incubated with CFTRinh172 alone (HTS + 172, n = 59 beads from 12 tracheas; HTS + Bumet + NA + 172 + HCO_3_^−^-free, n = 24 beads from 7 tracheas; ITS + Bumet + NA + 172 + HCO_3_^−^-free, n = 18 beads from 7 tracheas). Data are presented as mean ± SEM and values at 18 min were analyzed with ANOVA and Tukey’s multiple comparison test. Data sets labeled with different letters differ significantly, p < 0.05.

HTS treatment of both wild-type preparations incubated in the CFTR inhibitor CFTRinh172 as well as CFTR^−/−^ tracheas (Fig. 4H), resulted in an increased in ASL height. This suggests that HTS may trigger ASL production by the airway epithelia in a CFTR-independent manner (Fig. 5A). The CFTR-independent ion transport-related effect of HTS was blocked in wild-type preparations incubated in the cocktail of blockers (i.e. CFTRinh172, bumetanide, and niflumic acid in HCO_3_^−^-free bath, Fig. 5A). In contrast, ASL height in ITS-treated preparations was inhibited similarly by CFTR inhibitor alone and by the ion blocker cocktail (Fig. 5B), indicating that the blocker cocktail specifically blocks the effect of HTS treatment. These results suggest that the CF airway epithelium responds to HTS treatment with active ASL secretion independent of CFTR.

To estimate the proportion of ASL produced by active ASL secretion by CF epithelia, we incubated preparations in CFTRinh172, bumetanide, and niflumic acid in HCO_3_^−^-free saline and treated them with HTS or ITS (Fig. 5C, Supplementary Table 2). Since these preparations could not produce ASL through epithelial secretion, the difference in ASL produced by HTS and ITS treated preparations must have been driven by the osmotic effect alone, generated by the hypertonic treatment. Incubation with HTS + Bumet + NA + 172 + HCO_3_^−^ increased ASL height by 9.14 ± 0.69 µm. ITS + Bumet + NA + 172 + HCO_3_^−^ treatment produced an increase in ASL height of 4.23 ± 0.5 µm. HTS + 172 treatment increased ASL height by 13.32 µm. Since tissues incubated in Bumet + NA + 172 + HCO_3_^−^ cannot produce active secretion of ASL, the difference between HTS treatment (9.14 µm) and ITS (4.23 µm) can only be the result of osmotic effect (due to the exclusion of active epithelial secretion). Thus, the ASL height produced by osmotic effect alone is 4.96 µm. The ASL produced by osmotic effect plus active secretion in a CF model is 9.09 µm, the difference between HTS + 172 and ITS + Bumet + NA + 172 + HCO_3_^−^. Hence, the osmotic effect alone, 4.96 µm, is 54% of the osmotic plus active epithelial secretion, 9.09 µm. The results suggest that ~50% of the ASL produced by HTS treatment in CF is generated by active ASL secretion by airway epithelia.

## Discussion

The key finding in this study is that nebulized HTS treatment causes ASL production through the stimulation of the nervous system, which triggers active ASL secretion by airway epithelia. This pathway occurs alongside the osmotic effect of HTS that draws water from the serosal surface into the ASL^12^. The presence of hypertonic saline may be detected by Aδ- and C-fibers^16,17^, probably as a change in osmolality or ion concentration in the sensory neurons^16^. The local release of neurotransmitters may stimulate ASL secretion from airway submucosal glands^36^, and possibly modulate surface airway epithelia ion transport^41^. Aδ- and C-fibers are more frequent in the larger airways^21^, which may explain the recent unexpected finding that HTS treatment has a stronger effect on mucociliary clearance in larger airways than in small ones^10^.

In our *ex vivo* preparations, we detected substance P- and cholinergic-mediated stimulation of ASL secretion. However, only cholinergic stimulation had an effect on preparations without functional CFTR, suggesting that the contribution of the nervous system on HTS-triggered ASL secretion is different in CF and non-CF airways^18^. Our results indicate that it may be possible to modulate the duration and intensity of HTS treatment by pharmacologically modulating the contribution of the nervous system to HTS-triggered ASL secretion, which provides a potential target for future drug development.

Finally, our results highlight that the use of HTS formulations which include blockers of epithelial ASL reabsorption (e.g. ENaC blockers) may reduce the effect of HTS treatment if they interfere with epithelial ASL secretion by altering the epithelium physiology or through non-specific effects on other ion transporters^1^. The involvement of active ASL secretion by the airway epithelia in HTS-triggered ASL production suggests the possibility that in addition to improving mucociliary clearance, hypertonic saline may also increase the production of epithelia-secreted molecules, such as mucin and antimicrobial compounds, that contribute to airway sterility.

## Methods

### Animals

Female and male wild-type pigs were purchased from the Prairie Swine Centre, University of Saskatchewan. One-week old (~3 kg) piglets were used for *in vivo* imaging; ~5-weeks-old juvenile pigs (~15 kg) were used for isolated trachea experiments. In addition, for *ex vivo* airway imaging, CFTR^−/−^ tracheas were obtained from 6 newborn (6 to 12 h after birth), gut-corrected CFTR knockout pigs (*CFTR*^−/−^;*TgFABP* > *pCFTR* pigs), purchased from Exemplar Genetics (Exemplar Genetics, Sioux Center, IA, USA). The tracheas were dissected within 30 minutes of euthanasia, clamped at both ends to prevent liquid entering the lumen and placed in ice-cold Krebs-Ringer saline solution^23^. For the bicarbonate-free Krebs-Ringer saline solution, 25 mM NaHCO_3_ was replaced with 24 mM NaCl and 1 mM HEPES, pH 7.4, equilibrated in O_2_^42^. Both solutions had similar osmotic pressures of 270 ± 0.7 and 269 ± 0.5 (n = 6).

### Synchrotron-based x-ray imaging set-up

We used a novel synchrotron-based imaging method to quantify ASL secretion in the airway^23,24^. Experiments were performed using the BioMedical Imaging and Therapy-Bending Magnet (BMIT-BM) beamline 05B1-1, at the Canadian Light Source (CLS), Saskatchewan, Canada. The experimental hutch is located 25.5 meters away from the storage ring. Phase contrast imaging (PCI) was performed using monochromatic 33.4 KeV (λ = 0.37 nm) x-ray for live pig imaging and 20 keV (λ = 0.062 nm) x-rays for isolated trachea imaging, selected using a standard double-crystal monochromator. The beam size was 100.0 mm (wide) x 8.0 mm (vertical). The distance between the sample and the detector was 100 cm (for *in vivo* imaging) and 65 cm (for *ex vivo* isolated trachea imaging). Images were captured using a Fiber Optic Camera (C4742-56-12HR, Hamamatsu Photonics, San Jose, CA, USA) or a high-resolution x-ray converter (AA-60, Hamamatsu Photonics, San Jose, CA, USA) with a charge-coupled device (CCD) detector (C9300-124, Hamamatsu Photonics, San Jose, CA, USA). The pixel size of the image was 11.2 × 11.2 μm (Fiber Optic Camera) or 8.75 × 8.75 μm (CCD camera). Exposure time ranged from 500 to 900 ms.

### Agarose bead preparation

We used agarose beads as ‘measuring rods’ to determine the height of the ASL layer, which was measured as the distance between the air/ASL interface and the edge of the agarose bead touching the surface epithelia, as described in detail elsewhere^24^. Agarose beads were made in sterile conditions with 4% agarose in PBS as described^23,24^. All solutions used in the process of making agarose beads were autoclaved at 250 °C for at least 20 min. Warm (50–55 °C) 4% agarose solution was made in PBS. The agarose solution was mixed with warm (50–55 °C) heavy paraffin oil and stirred rapidly, achieving a vortex 2 cm in depth. The agarose/oil mixed solution was left in a beaker for 11 min, and then ice was slowly added around the beaker for 7 min. After 7 min of cooling, the agarose/oil solution was poured into a separatory funnel containing warm (50–55 °C) 0.5% w/v sodium deoxycholate in PBS to wash the mineral oil from the beads. The agarose beads were allowed to settle and then washed three times with PBS at room temperature to wash away the sodium deoxycholate. The upper one third of agarose beads at the bottom of the separatory funnel were then taken and used for the experiments. Agarose beads loaded with lidocaine, and capsaicin were prepared by mixing chemicals with warm (50–55 °C) 4% agarose solution right before gelation procedure.

Preliminary experiments showed that beads are not visible unless we added a contrast agent. Thus, in order to make the beads visible by x-ray, we added BaSO_4_ (nominal 1 M) as contrast agents to the PBS. This salt was chosen because it is insoluble in water and does not contribute to the osmotic pressure of the bead. The osmolarity of the beads solutions were 278 ± 0.1 and 276 ± 0.3 for PBS and PBS plus 1 M BaSO_4_, respectively^23^.

### Experimental set-up

For *in vivo* swine imaging, the animal was held in supine position and fitted with a mask connected to an anesthetic machine providing 2% isoflurane in pure medical oxygen at 1 l/min (Fig. 1A). Throughout the experiment, the animals breathed spontaneously. Respiratory rate, heart rate, body temperature, and O_2_ saturation level as well as the plane of anesthesia were monitored. The larynx was sprayed with lidocaine to prevent a reflex response in preparation for intubation for every experiment. An endotracheal tube was placed at the opening of the larynx into the trachea and the agarose beads (~400 to 1200 μm in diameter)^24^ were blown through the endotracheal tube into the trachea with a puff of air, after which, the endotracheal tube was immediately removed.

For *ex vivo* airway imaging, a trachea was clamped at both ends to prevent contamination of the lumen with blood or other fluids. The cartilage was removed with a scalpel and a fine blunt-ended elevator to improve access of the drugs to the epithelia and nervous tissue. The trachea preparation was placed in a custom-built chamber. The tissue was immersed in Krebs solution plus 1 μM indomethacin at 35 °C and equilibrated with 95% O_2_ and 5% CO_2_. The lumen of the trachea preparation remained free of solution and sealed to keep the lumen from desiccating, yet it could be opened and accessed by the researchers to introduce the agarose beads. Agarose beads (~400 to 1000 μm in diameter) were blotted dry and placed in the lumen of the preparation using a cotton swab^23^. To test the effect of airway neurons and epithelia on HTS treatment, atropine, tetrodotoxin, CFTRinh172, NK-1 blocker L-703606, bumetanide, and niflumic acid were dissolved in Krebs solution using in the custom-built chamber.

Each bead was placed at a location where the air/ASL interface was parallel to the x-rays penetrating the sample, i.e. the top or bottom of the preparation^23,24^, using a motorized computer-controlled experimental stage to rotate the preparation (i.e. isolated preparation and live animal). Hypertonic (7% NaCl solution w/v) and isotonic (0.9% NaCl solution w/v) saline treatments were administered using a nebulizer (705-445, AMG Medical Inc, Montreal, Quebec, Canada), which produces liquid aerosols with a median diameter of 4 μm. Nebulization was performed for a period of 90 s with delivery of a total amount of 0.3 ml of liquid. For *in vivo* experiments, nebulized liquid was directly delivered into the face mask covering the swine. For *ex vivo* experiments, aerosol was delivered through one end of the isolated trachea with the other end opened to allow excess aerosol to flow out of the preparation. Hypertonic saline or isotonic saline treatment began at time 0, and the ASL volume increased was tested 18 min after treatment. Images were captured 3 minutes before treatment (−3 min), and 6, 12, and 18 minutes after treatment (Fig. 1C).

### ASL height measurement

We exploited the large refractive index difference between the air and the ASL layer, which produces a strong signal at the air/ASL interface, using phase contrast imaging (PCI) (Fig. 1B)^23–25,43,44^. Because PCI cannot delineate the ASL/tissue interface, we established the position of the tissue with respect to the air/ASL interface using agarose beads as “measuring rods”. The agarose beads are placed in the ASL and come into direct contact with the surface epithelium due to the force generated by the surface tension of the ASL, as shown elsewhere^24^. The surface tension of the ASL immobilizes the beads on the surface of the epithelium (i.e. beads are not cleared away by airway cilia), and the liquid secreted by the airway, which would normally be cleared from the airway due to mucociliary clearance, is retained around the static bead, allowing us to measure the accumulation of the ASL produced by fluid secretion^23,24^. In a small number of cases, 1 in 29 of the *in vivo* experiments and 3 in 70 of the *ex vivo* experiments, the airway displayed a change in diameter during the experiment. Since this could affect our measurements, we removed those experiments from our data set to eliminate any possible artefacts that may confound the interpretation of the data.

A researcher blinded to the experimental conditions measured the height of the ASL layer as the distance between the air/ASL interface and the edge of the agarose bead touching the surface epithelium (Fig. 1B)^23,24^. The data is presented as the difference in ASL height (Δ Liquid layer) at every time point and the initial measurement 3 min before nebulization (time −3 min).

### Statistics

To test the effect of each experimental condition, we compared the ASL height produced by each preparation at 18 min using ANOVA and Tukey’s multiple comparisons tests in GraphPad Prism 5 (GraphPad Software Inc., San Diego, CA, US), with p < 0.05 considered significant. Data are presented as mean ± S.E.M, where each individual agarose bead is a data point^23,24^.

The data sets labeled HTS and ITS in Fig. 1D are the same as those in Fig. 2A,B. Data sets labeled HTS and ITS in Fig. 1E,F are the same as those in Fig. 3A,B and D; and Fig. 4A,B. Data sets labeled HTS + 172, and ITS + 172 in Fig. 3B are the same as those in Fig. 3C,E and G; and Fig. 4A,B and C.

### Reagents

Drugs were obtained from Sigma-Aldrich unless otherwise stated. CFTRinh172 was purchased from Cedarlane Labs (Burlington, ON, CA), tetrodotoxin was obtained from Alomone labs (Jerusalem, Israel), and lidocaine hydrochloride spray was acquired from Odan Laboratories LTD (Montreal, Canada). Stock solutions of CFTRinh172, lidocaine, atropine, bumetanide, niflumic acid, and L-703606 were dissolved in DMSO. The final concentration of DMSO was less than 0.1%. Tetrodotoxin was directly dissolved into purified water.

### Study approval

All experiments were performed with the approval of the Canadian Light Source and the Animal Ethics Committee at the University of Saskatchewan. All experiments were performed in accordance with relevant guidelines and regulations established by the Animal Ethics Committee at the University of Saskatchewan and the Canadian Council on Animal Care.

## Electronic supplementary material

Supplementary data

## Data Availability

All data generated or analysed during this study are included in this published article.
