# Supplementary material for: Nebulized hypertonic saline triggers nervous system-mediated active liquid secretion in cystic fibrosis swine trachea
Source: Sci Rep. 2019 Jan 24;9:540. doi: 10.1038/s41598-018-36695-4 (PMC6345831; doi:10.1038/s41598-018-36695-4)
Supplement: Supplementary file 1 — Supplementary data [file 41598_2018_36695_MOESM1_ESM.pdf]

Title: Nebulized hypertonic saline triggers nervous system-mediated active liquid secretion in cystic fibrosis swine trachea

Authors and affiliations

Xiaojie Luan[1], Julian S. Tam[2], George Belev[3], Santosh Jagadeeshan[1], Brendan Murray[1], Noman Hassan[1], Terry E. Machen[4], L. Dean Chapman[5], and Juan P. Ianowski[1]

1. University of Saskatchewan, Department of Physiology, Health Science Building, Room 2D01, 107 Wiggins Rd., Saskatoon, Saskatchewan, S7N 5E5, Canada.
2. University of Saskatchewan, Department of Medicine, Division of Respirology, Critical Care, and Sleep Medicine. Royal University Hospital, 103 Hospital Drive, Saskatoon, Saskatchewan, S7N 0W8, Canada
3. Canadian Light Source Inc., 44 Innovation Boulevard, Saskatoon, Saskatchewan, S7N 2V3, Canada.
4. University of California, Department of Molecular and Cell Biology, 231 LSA, Berkeley, CA, 94720-3200
5. University of Saskatchewan, Department of Anatomy and Cell Biology, Health Science Building, Room 2D01, 107 Wiggins Rd., Saskatoon, Saskatchewan, S7N 5E5, Canada.

Table 1: ASL measurements in wild-type swine *in vivo*

Group	ASL layer height (µm) Mean ± SEM						
	Initial (-3 min)	6 min	Δheight (-3 to 6 min)	12 min	Δheight (-3 to 12 min)	18 min	Δheight (-3 to 18 min)
HTS n=6	142.29 ± 30.66	165.29 ± 23.53	23.00 ± 10.92	197.42 ± 27.88	55.13 ± 9.63	237.25 ± 26.12	94.96 ± 18.56
ITS n=6	176.83 ± 29.93	188.54 ± 33.93	11.71 ± 5.07	198.42 ± 35.22	21.58 ± 6.59	210.83 ± 34.30	34.00 ± 5.58
HTS+Atro+Lido n=6	180.13 ± 26.42	192.58 ± 30.43	12.46 ± 7.22	220.42 ± 30.90	40.29 ± 9.36	242.38 ± 28.50	62.25 ± 8.65
ITS+Atro+Lido n=5	161.45 ± 30.44	165.85 ± 29.07	4.40 ± 2.69	171.55 ± 29.92	10.10 ± 3.52	186.05 ± 30.40	24.60 ± 6.66
HTS+Capsaicin n=6	121.00 ± 20.93	152.50 ± 18.96	31.50 ± 10.97	192.67 ± 27.93	71.67 ± 19.94	228.13 ± 25.64	107.13 ± 15.02
ITS+Capsaicin n=5	118.10 ± 35.30	135.65 ± 38.05	17.55 ± 5.06	151.00 ± 38.82	32.90 ± 6.44	181.25 ± 34.05	63.15 ± 4.59

Table 2: ASL measurements in wild-type swine *ex vivo*

Group	ASL layer height (µm) mean ± SEM						
	Initial (-3 min)	6 min	Δheight (-3 to 6 min)	12 min	Δheight (-3 to 12 min)	18 min	Δheight (-3 to 18 min)
HTS<br>n=45	93.61<br>± 5.83	99.42<br>± 5.97	5.81<br>± 0.63	105.53<br>± 5.78	11.92<br>± 0.58	112.53<br>± 5.80	18.92<br>± 0.74
ITS<br>n=49	84.14<br>± 4.17	86.70<br>± 4.28	2.55<br>± 0.37	91.46<br>± 4.27	7.32<br>± 0.46	95.54<br>± 4.25	11.39<br>± 0.59
Control<br>n=12	71.53<br>± 7.60	73.65<br>± 7.56	2.12<br>± 0.41	79.33<br>± 7.41	7.80<br>± 0.46	82.76<br>± 7.34	11.23<br>± 0.81
HTS+Amiloride<br>n=12	100.77<br>± 15.48	106.60<br>± 15.81	5.83<br>± 1.65	111.71<br>± 15.62	10.94<br>± 1.14	119.73<br>± 14.83	18.96<br>± 1.81
HTS+Lido+TTX<br>n=18	100.50<br>± 5.97	105.36<br>± 5.70	4.86<br>± 1.06	108.27<br>± 5.55	7.78<br>± 1.20	113.13<br>± 5.99	12.64<br>± 1.06
ITS+Lido+TTX<br>n=9	109.47<br>± 17.03	112.39<br>± 17.43	2.92<br>± 1.46	115.31<br>± 17.54	5.83<br>± 1.46	119.19<br>± 16.91	9.72<br>± 1.75
HTS+172<br>n=59	89.56<br>± 4.33	93.37<br>± 4.35	3.81<br>± 0.44	97.67<br>4.29	8.11<br>± 0.46	102.88<br>± 4.30	13.32<br>± 0.50
ITS+172<br>n=29	85.58<br>± 5.49	87.39<br>± 5.46	1.81<br>± 0.55	88.77<br>± 5.49	3.20<br>± 0.62	90.71<br>±5.57	5.13<br>± 0.63
HTS+172+Lido+TTX<br>n=9	88.21<br>± 8.11	91.13<br>± 8.46	2.92<br>± 1.46	93.07<br>± 9.20	4.86<br>± 1.54	95.99<br>± 8.00	7.78<br>± 0.97
ITS+172+Lido+TTX<br>n=21	86.67<br>± 6.83	88.75<br>± 6.76	2.08<br>± 0.83	90.83<br>± 6.63	4.17<br>± 0.98	92.50<br>± 6.52	5.83<br>± 1.10
HTS+NK-1 blocker<br>n=36	112.79<br>± 10.48	118.23<br>± 10.40	5.44<br>± 0.60	123.33<br>± 10.51	10.55<br>± 1.07	128.17<br>± 10.75	15.39<br>± 1.16
ITS+NK-1 blocker<br>n=16	71.48<br>± 7.46	75.30<br>± 7.37	3.83<br>± 1.12	80.77<br>± 7.38	0.30<br>± 0.70	83.51<br>± 7.75	12.03<br>± 1.57
HTS+172+NK-1 blocker<br>n=10	88.73<br>± 7.74	91.35<br>± 7.74	2.63<br>± 1.34	96.60<br>± 7.56	7.88<br>± 0.88	101.85<br>± 7.58	13.13<br>± 1.96
ITS+172+NK-1 blocker<br>n=17	113.75<br>± 13.61	115.29<br>± 13.68	1.54<br>± 0.83	117.35<br>± 13.86	3.60<br>± 1.08	119.41<br>± 13.94	5.66<br>± 1.05
HTS+Atropine<br>n=22	104.71<br>± 6.71	108.29<br>± 6.71	3.58<br>± 0.94	112.66<br>± 6.63	7.96<br>± 1.14	117.44<br>± 6.94	12.73<br>± 1.49
ITS+Atropine<br>n=11	96.93<br>± 10.42	100.90<br>± 11.36	3.98<br>± 1.38	105.68<br>± 11.12	8.75<br>± 1.18	109.65<br>± 10.98	12.73<br>± 1.38
HTS+172+Atropine<br>n=18	98.02<br>± 6.92	100.93<br>± 7.23	2.92<br>± 1.00	104.34<br>± 6.66	6.32<br>± 1.19	107.25<br>± 6.71	9.24<br>± 1.32
ITS+172+Atropine<br>n=19	92.11<br>± 9.13	93.03<br>± 9.28	0.92<br>± 0.63	95.33<br>± 9.67	3.22<br>± 1.00	97.63<br>± 9.40	5.53<br>± 1.20
HTS+Bumet+NA+172+HCO_3^--free<br>n=24	97.34<br>± 4.99	99.71<br>± 4.93	2.37<br>± 0.56	103.21<br>± 5.01	5.87<br>± 0.72	106.53<br>± 5.05	9.19<br>± 0.69
ITS+Bumet+NA+172+HCO_3^--free<br>n=18	120.90<br>± 12.78	122.16<br>± 12.81	1.26<br>± 0.20	123.67<br>± 12.80	2.77<br>± 0.29	125.13<br>± 12.77	4.23<br>± 0.50

Table 3: ASL measurements in CFTR$^{-/-}$ swine *ex vivo*

Group	ASL layer height (μm) Mean ± SEM						
	Initial (-3 min)	6 min	Δheight (-3 to 6 min)	12 min	Δheight (-3 to 12 min)	18 min	Δheight (-3 to 18 min)
HTS<br>n=6	226.04<br>± 77.65	234.79<br>± 80.18	8.75<br>± 5.53	261.04<br>± 78.08	35.00<br>± 7.14	268.33<br>± 78.25	42.29<br>± 8.28
ITS<br>n=4	144.38<br>± 16.17	146.56<br>± 15.72	2.19<br>± 2.19	148.75<br>± 15.16	4.38<br>± 2.53	155.31<br>± 19.03	10.94<br>± 4.19
HTS+Lido+TTX+Atro<br>n=4	137.81<br>± 32.03	142.19<br>± 29.54	4.38<br>± 2.53	148.75<br>± 30.52	10.94<br>± 2.19	161.88<br>± 28.24	24.06<br>± 5.51
